# Germacrone alleviates okadaic acid-induced neurotoxicity in PC12 cells via M1 muscarinic receptor-mediated Galphaq (Gq)/phospholipase C beta (PLCβ)/ protein kinase C (PKC) signaling

**DOI:** 10.1080/21655979.2022.2036918

**Published:** 2022-02-14

**Authors:** Mingqin Lin, Peiqiong Li, Wei Liu, Tianqi Niu, Liping Huang

**Affiliations:** a School of Basic Medicine and Life Science, Hainan Medical University, Haikou, Hainan, China; b School of Chemistry and Chemical Engineering, Lingnan Normal University, Zhanjiang, Guangdong, China

**Keywords:** Alzheimer’s disease, Germacrone, CHRM1, Gq/PLCΒ/PKC signaling, oxidative stress

## Abstract

Alzheimer’s disease (AD) is a neurodegenerative disorder with prominent individual morbidity and mortality among elderly people. Germacrone (Germ) has been reported to exert dominant protective roles in multiple human diseases, and neurological diseases are also included. The intention of this paper is to determine the impacts of Germ on okadaic acid (OA)-treated PC12 cells and confirm the hidden regulatory mechanism. First, PC12 cells were induced by OA in the absence or presence of Germ. Cell counting kit-8 assay was to monitor cell proliferation. Western blot was to test the protein levels of cholinergic muscarinic M1 receptor (CHRM1), Galphaq (Gq), phospholipase C beta (PLCβ) and protein kinase C (PKC). The levels of reactive oxygen species (ROS) and other oxidative stress markers were evaluated using corresponding kits. ELISA was used to estimate the levels of AD markers. RT-qPCR was used to examine the mRNA levels of beta-site amyloid-precursor-protein-cleaving enzyme 1 (BACE-1) and apolipoprotein E (APOE). The results uncovered that Germ enhanced the proliferation of OA-insulted PC12 cells, elevated the protein level of CHRM1 and activated the Gq/PLCβ/PKC signaling. Moreover, after OA-induced PC12 cells were administered with Germ, insufficiency of CHRM1 impeded cell proliferation, enhanced oxidative stress and neuron injury and inactivated the Gq/PLCβ/PKC signaling. Furthermore, the addition of Gq inhibitor UBO-QIC, PLCβ inhibitor U73122 or PKC inhibitor Go6983 reversed the enhanced proliferation, the reduced oxidative stress and neuron injury in OA-treated PC12 cells caused by Germ. Collectively, Germ modulated M1 muscarinic receptor-mediated Gq/PLCβ/PKC signaling, thereby alleviating OA-induced PC12 cell injury.

## Graphical Abstract

**Figure:**
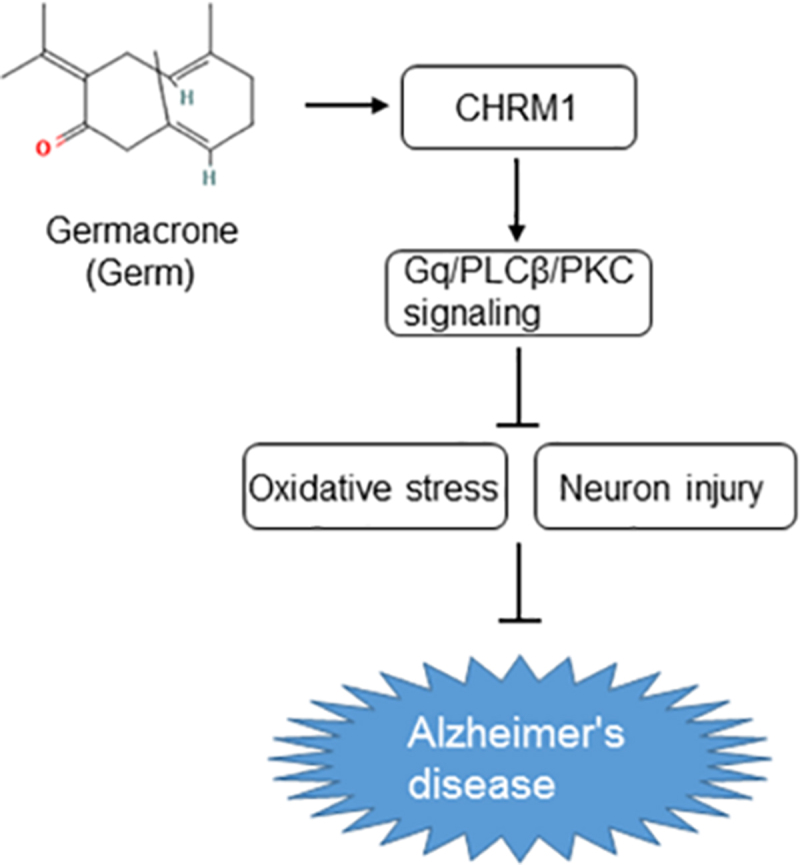

## Introduction

Dementia refers to a kind of brain disease featured by the long-term and gradual cognitive impairment [1]. As the most frequent type of dementia, Alzheimer’s disease (AD) takes up 60–80% of all the cases [2]. The occurrence of AD is increasingly high, in part due to the growing aging population [3]. Risk factors including aging, heredity, environmental factors, infections, head injuries and vascular diseases have been documented to be implicated in the onset of AD [4]. Moreover, cholinergic and amyloid hypotheses have been considered as the main causes of AD [4]. Cholinesterase inhibitors and memantine have been acknowledged as approved drug treatments for AD, while anti-amyloid therapy, immunization, monoclonal antibodies and tau-targeted therapy are considered as potential future drug treatments [5]. However, there is no successful clinical trial with a novel therapy based on amyloid cascade and tau protein hypotheses [6]. Therefore, the possible mechanism under the pathology of AD still needs further exploration.

**Figure 1.**
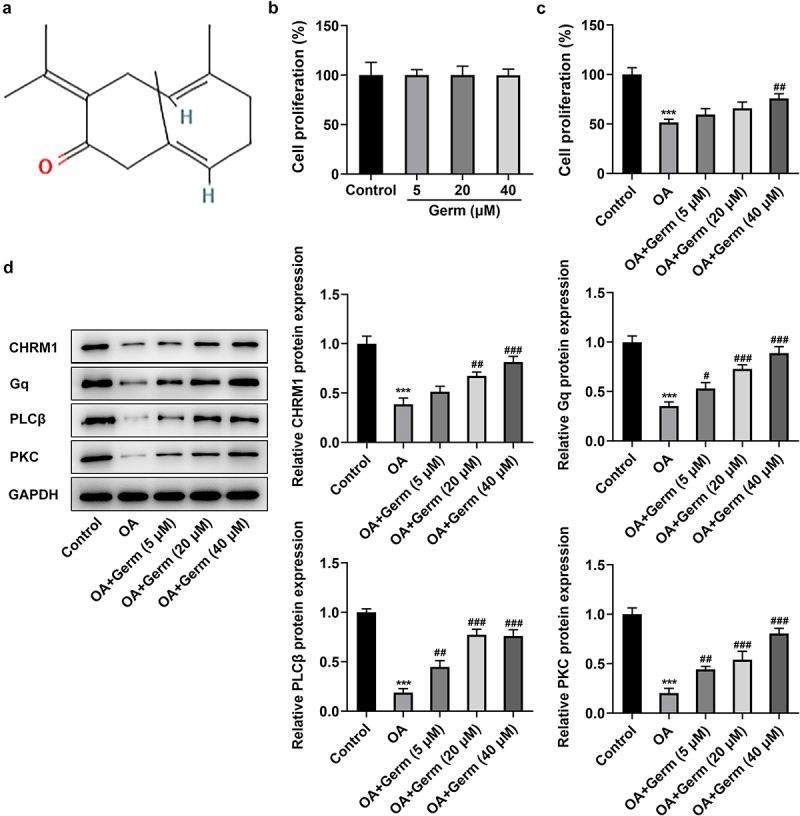
Germ elevates the proliferation, upregulates CHRM1 expression and activates the Gq/PLCβ/PKC signaling in OA-treated PC12 cells. (a) The chemical structure of Germ. (b) CCK-8 assay appraised the proliferation of PC12 cells after administration of different concentrations of Germ. (c) The impacts of Germ on the proliferation of OA-treated PC12 cells were assessed by CCK-8 assay. (d) Western blot was used to detect the protein levels of CHRM1, Gq, PLCβ and PKC. ***P < 0.001 vs. control; ^#^P < 0.05, ^##^P < 0.01, ^###^P < 0.001 vs. OA.

Germacrone (Germ) (Figure 1(a)) is one of the key components found in the extracted Chinese medicine zedoary turmeric [7]. It has been reported that Germ elicits anti-inflammatory, antioxidant, antiviral and neuroprotective activities [8]. For instance, Germ plays an anti-oxidative role to mitigate cerebral ischemia/reperfusion injury [9]. Moreover, curcuma zedoaria extract may alleviate cognitive impairment in pentylenetetrazol-stimulated epilepsy rats [10]. Germ is capable of ameliorating neuroinflammation and oxidative stress in traumatic brain injury [11]. More importantly, Germ hampers PC12 cell autophagy to ease oxygen–glucose deprivation/reperfusion injury [12]. The PC12 cell model is a useful model to study cellular signal transduction, neurodegenerative disease, which has been widely used in the investigation about anti-Alzheimer’s and anti-aging drug development [13–15]. Nevertheless, it is urgent to confirm the hypothesis that Germ may possess neuroprotective property in AD.

It is defined that muscarinic acetylcholine receptors (mAChRs) consist of a subfamily of G protein-coupled receptors that are widely expressed and serve as vital modulators in the central and peripheral nervous system [16,17]. Among these, M1 muscarinic receptor is the most significant subtype. Yin et al. have proposed that M1 muscarinic receptor boosts the migration and invasion of prostate cancer cells [18]. M1 muscarinic receptor contributes to pulmonary and hepatic injury in LPS-triggered endotoxemia mice model [19]. On the contrary, the activation of M1 muscarinic receptor has been revealed to treat cognitive deficits and alter the occurrence or development of AD dementia [20]. Also, it has emerged as a pivotal therapeutic target for AD [21]. Intriguingly, turmeric, from which Germ is discovered, elevates the expression of M3, M5 cholinergic muscarinic receptors and enhances learning and memory in stress-induced mouse model [22]. Additionally, M1 mAChRs (CHRM1) are classically coupled to Galphaq (Gq) family to trigger the activation of phospholipase C beta (PLCβ) and protein kinase C (PKC) to decrease Aβ and tau hyperphosphorylation [23,24]. CHRM1 also plays a neuroprotective role in PC12 cells via PKC-mediated ERK1/2 phosphorylation [25].

The purpose of this study is to confirm the influences of Germ on okadaic acid (OA)-treated PC12 cells and determine the correlation between Germ and M1 muscarinic receptor-mediated Gq/PLCβ/PKC signaling in AD. Our findings might offer a novel clue for the therapeutic strategy of AD.

## Materials and methods

### Cell culture and treatment

Rat PC12 cells procured from American Type Culture Collection (ATCC; Manassas, VA) were grown at Roswell Park Memorial Institute (RPMI) 1640 medium (Gibco, Gaithersburg, MD, USA) with 10% fetal bovine serum (FBS; Biologic Industries, Beit Haemek, Israel) as well as 1% antibiotics (Sigma-Aldrich, St. Louis, MO) and placed in a humidified incubator with 5% CO_2_ at 37°C. For further induction, PC12 cells were exposed to 175 nM OA (Sigma-Aldrich, St Louis, MO) for 48 h [26], following pretreatment with 5, 20 and 40 µM concentrations of Germ (Shanghai YuanYe Biotechnology Co., Ltd.) at 37°C for 0.5 h. Germ was dissolved in dimethylsulfoxide (DMSO; Sigma-Aldrich, St Louis, MO), and the final concentration of DMSO was no more than 0.1%. PC12 cells in the control group were treated with 0.1% DMSO. In addition, PC12 cells were cultivated with Gq inhibitor UBO-QIC (100 nM; E. Kostenis, University of Bonn, Germany) [27], PLCβ inhibitor U73122 (10 μM; Sigma-Aldrich, St. Louis, MO) [28] or PKC inhibitor Go6983 (10 μM/ml; Sigma-Aldrich, St. Louis, MO) [29] to validate that the neuroprotective effects of Germ on PC12 cells were regulated by the Gq/PLCβ/PKC signaling.

### Plasmid transfection

For transfection, to avoid off-target effect, two short hairpin RNAs (shRNAs) targeting CHRM1 (sh-CHRM1#1/2) designed from different open reading frame (ORF) regions and the negative control (sh-NC) was generated by Shanghai GenePharma. The shRNA sequences were as follows: sh-CHRM1#1, 5’-GCAACCTACTGGTACTCATCT-3’; sh-CHRM1#2, 5’-GCACCTTCTCCATGAACCTCT-3’; sh-NC, 5’-TTCTCCGAACGTGTCACGT-3’. The indicated plasmids were transfected into PC12 cells at 80% confluence by means of lipofectamine 3000 reagent (Life Technologies) following the manufacturer’s protocol. At 48 h after post-transfection, the transfection efficiency was verified.

### Cell counting kit-8 (CCK-8)

CCK-8 kit (Abmole Bioscience, Houston) was adopted to detect cell proliferation in conformity to the product manual. After treatment or transfection, these cells were inoculated into a 96-well plate and incubated at 37°C. Subsequently, the OD450 value for each well was monitored by the use of a microplate reader (Dynex Technologies, West Sussex, UK) after the addition of 10 μl CCK-8 solution.

### Reverse transcription-quantitative PCR (RT-qPCR)

Total RNA preparation from PC12 cells and the conversion of RNA to complementary DNA (cDNA) were separately executed by virtue of TRIzol (Ambion, USA) and PrimeScript RT reagent kit (Takara, Kyoto, Japan). qPCR was performed using a one-step RT-PCR kit (Takara Bio, Inc.) in a 7500 Real Time PCR System (Applied Biosystems®) on the basis of the manufacturer’s instructions. The thermocycling conditions were as follows: denaturation at 95°C for 5 min, followed by 40 cycles of 95°C for 10 sec and 60°C for 30 sec. The primer sequences used in this study were shown as follows: CHRM1: forward, 5’-TGGCTGGTTTCCTTCGTTCT-3’, reverse, 5’-GCTTCAGAATCTACCATGGGC-3’; beta-site amyloid-precursor-protein-cleaving enzyme 1 (BACE-1): forward, 5’-GTTTGTCACGGCAGACATGG-3’, reverse, 5’-TGCTTTCTCCCTCCTGTTTCC-3’; apolipoprotein E (APOE): forward, 5’-GCTCCCAAGTCACACAGGAA-3’, reverse, 5’-CGGTTGCGTAGATCCTCCAT-3’; glyceraldehyde-phosphate dehydrogenase (GAPDH): forward, 5’-TGTGAACGGATTTGGCCGTA-3’, reverse, 5’-GATGGTGATGGGTTTCCCGT-3’. The relative mRNA levels were calculated using the 2^−∆∆Cq^ method [30].

### Western blot

Cells were subjected to protein isolation using radioimmunoprecipitation (RIPA) buffer (Beyotime, Shanghai, China). The protein concentration was then determined using a bicinchoninic acid (BCA) protein assay kit (Pierce, Rockford, IL, USA). Proteins were fractionated by 10% sodium dodecyl sulfate-polyacrylamide gel electrophoresis (SDS-PAGE). Following this, the separated proteins were moved to polyvinylidene difluoride (PVDF) membranes, impeded by 5% skimmed milk at 37°C, and cultivated with the primary antibodies at 4°C overnight. After three washes, the membranes were incubated with a horseradish peroxidase-linked secondary antibody (1:2,000; cat. no. ab97051; Abcam) at room temperature. Enhanced chemiluminescence (ECL) substrate kit (Tanon, Shanghai, China) was used to detect the bound antibodies, according to the manufacturer’s instructions, and Image-Pro Plus software (v6; Media Cybernetics, Inc., Rockville, MD, USA) was used to measure protein expression, referring to GAPDH as the internal control. Anti-CHRM1 (1:1,000; cat. no. BA1543) antibody was provided by BOSTER. Anti-Gq (1:1,000; cat. no. ab210004), anti-PLCβ (1:1,000; cat. no. ab250777), anti-PKC (1:2,000; cat. no. ab181558) and anti-GAPDH (1:10,000; cat. no. ab181602) antibodies were procured from Abcam.

### Estimation of reactive oxygen species (ROS)

To detect intracellular ROS, PC12 cells were plated into 24-well plates. PC12 cells were stained with 10 μΜ 2,7-Dichlorodi-hydrofluorescein diacetate (DCFH-DA; Sigma-Aldrich) at 37°C for half an hour in the dark and washed with serum-free RPMI 1640 medium three times. Finally, ROS staining was observed with the adoption of a fluorescence spectrometer (HTS7000, Perkin Elmer, USA).

### Detection of superoxide dismutase (SOD), glutathione peroxidase (GSH-Px) and malondialdehyde (MDA)

Cells were decomposed in 300 μl lysis buffer, and total protein was quantified by BCA kit (Pierce, Rockford, IL, USA). Corresponding commercial kits (Jiancheng Biotech, Nanjing, China) were used to determine the activities of SOD (A001-1-2), GSH-Px (A005-1-2) and MDA (A003-1-2) in accordance with the manufacturer’s guidance.

### Enzyme-Linked Immunosorbent Assay (ELISA)

With the employment of ELISA kits, the levels of AD biomarkers including phosphorylated tau (p-tau; cat. no. JL21148, Jianglaibio), amyloid beta-protein 42 (Aβ_42_; cat. no. ER0755, FineTest) and amyloid precursor protein (APP; cat. no. YPR107783, Ycextract) were estimated in the light of the standard protocol. The optical density values were read at 450 nm by a microplate reader (Dynex Technologies, West Sussex, UK).

### Statistical analyses

All data from the three independent experiments were denoted as the mean ± standard deviation and analyzed by GraphPad Prism (version 8.0; GraphPad Software, Inc.). Comparisons between the two groups were done by Student’s t-test and differences among other groups were estimated by one-way analysis of variance (ANOVA) with Tukey’s post hoc test. The values of P less than 0.05 were indicative of statistical significance

## Results

### Germ elevates the proliferation, upregulates CHRM1 expression and activates the Gq/PLCβ/PKC signaling in OA-induced PC12 cells

A considerable body of evidence indicates that Germ exerts neuroprotective effects in multiple diseases [9,11]. Whether Germ could alleviate the neuron injury during AD remains to be elucidated. Firstly, the effect of Germ on the proliferation of PC12 cells was assessed by CCK-8 assay and the results implied that following the treatment of different concentrations of Germ (5, 20 and 40 µM), the ability of PC12 cell proliferation had no significant difference compared with the control group (Figure 1(b)). However, the presence of OA prominently reduced the proliferation of PC12 cells and the addition of Germ enhanced cell proliferation under this condition (Figure 1(c)). Moreover, Western blot analysis revealed that PC12 cells exposed to OA exhibited lower protein levels of CHRM1, Gq, PLCβ and PKC relative to the control group, whereas the administration of different concentrations of Germ gradually raised the protein levels of CHRM1, Gq, PLCβ and PKC when compared to the OA group (Figure 1(d)). To sum up, Germ elevates the proliferation, upregulates CHRM1 expression and activates Gq/PLCβ/PKC signaling in PC12 cells in response to OA.

### Germ enhances the proliferation and reduces oxidative stress in OA-stimulated PC12 cells through activating CHRM1

**Figure 2.**
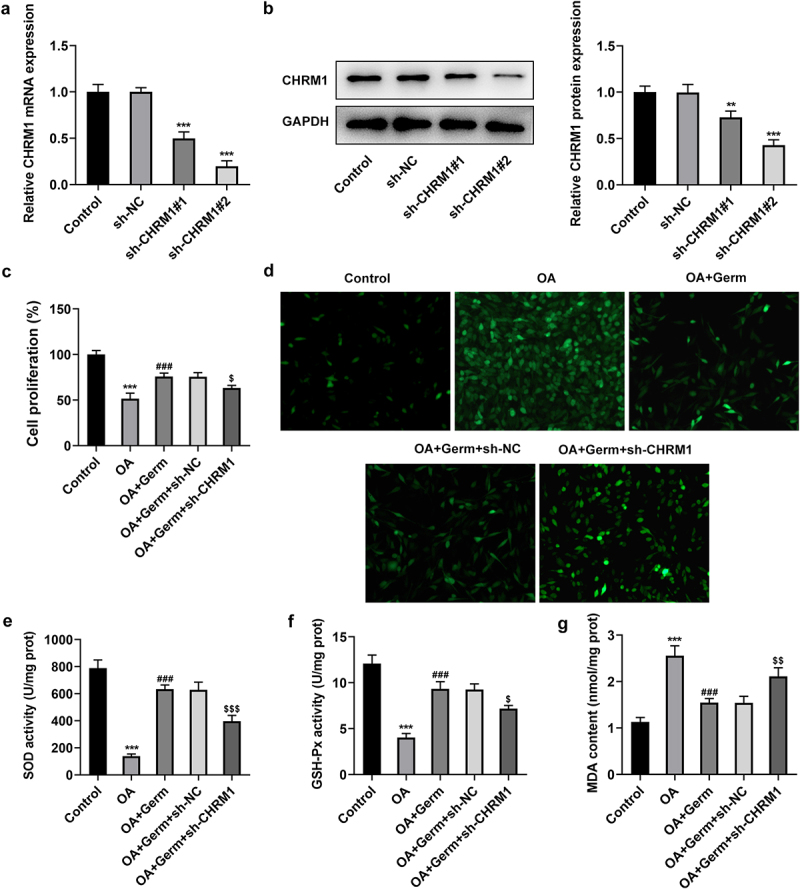
Germ enhances the proliferation and reduces oxidative stress in OA-stimulated PC12 cells through activating CHRM1. (a) RT-qPCR and (b) Western blot was employed to determine the knockdown efficiency of CHRM1. **P < 0.01, ***P < 0.001 vs. sh-NC. (c) CCK-8 assay was used to measure cell proliferation. (d) ROS level was estimated by DCFH-DA assay. (e) SOD, (f) GSH-Px and (g) MDA levels were examined by corresponding kits. ***P < 0.001 vs. control; ^###^P < 0.001 vs. OA; ^$^P < 0.05, ^$$^P < 0.01, ^$$$^P < 0.001 vs. OA+Germ+sh-NC.

Subsequently, CHRM1 was firstly silenced to validate whether Germ exerted its functions on OA-insulted PC12 cells via targeting CHRM1. It was discovered that CHRM1 expression dropped dramatically after transfection with sh-CHRM1#1/2 plasmids as compared to the sh-NC group (Figure 2(a-b)). Sh-CHRM1#2 was chosen for the following experiments owing to its better interference efficiency. CCK-8 assay manifested that the proliferative role of Germ in OA-treated PC12 cells was abrogated by down-regulation of CHRM1 (Figure 2(c)). Furthermore, the accumulation of ROS which is associated with oxidative stress [31] was investigated by DCFH-DA assay. It was noticed that ROS level was increased in PC12 cells in response to OA and Germ treatment greatly decreased ROS level, while this effect was reversed by CHRM1 insufficiency (Figure 2(d)). Additionally, SOD and GSH-Px levels were declined, while MDA level was elevated in OA-treated PC12 cells compared with the control group, while silencing of CHRM1 restored the up-regulated SOD and GSH-Px levels and the down-regulated MDA level imposed by Germ exposure (Figure 2(e-g)). In short, Germ up-regulates CHRM1 to promote the proliferation and ameliorate oxidative stress in OA-stimulated PC12 cells.

### Germ elevates CHRM1 expression to ease OA-elicited injury of PC12 cells

**Figure 3.**
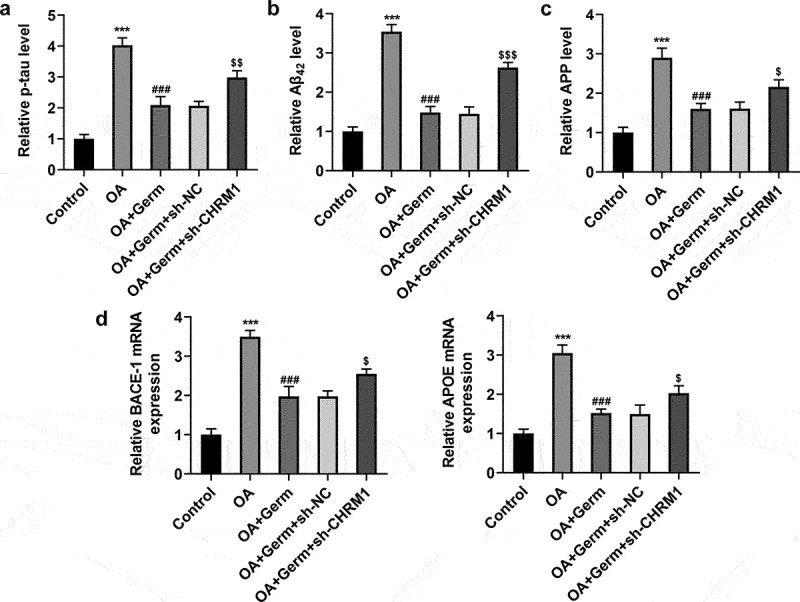
Germ elevates CHRM1 expression to ease OA-elicited injury of PC12 cells. (a) p-tau, (b) Aβ_42_ and (c) APP levels were detected by ELISA assay. (d) BACE-1 and APOE expression was tested by RT-qPCR. ***P < 0.001 vs. control; ^###^P < 0.001 vs. OA; ^$^P < 0.05, ^$$^P < 0.01, ^$$$^P < 0.001 vs. OA+Germ+sh-NC.

p-tau, Aβ_42_ and APP are core cerebrospinal fluid biomarkers for AD [32]. Whether Germ could regulate the expression of these genes to relieve the neuron injury was explored in the subsequent experiments. The levels of p-tau, Aβ_42_ and APP were analyzed by ELISA assay and results indicated that p-tau, Aβ_42_ and APP all exhibited higher levels in OA group in comparison with the control group. After PC12 cells were exposed to OA, the administration of Germ significantly cut down the levels of p-tau, Aβ_42_ and APP, while interference of CHRM1 partially offset this effect (Figure 3(a-c)). Similarly, BACE-1 and APOE expressions were examined by RT-qPCR analysis, and it was found that Germ decreased OA-triggered mRNA levels of BACE-1 and APOE in PC12 cells, while the inhibitory role of Germ was partially abrogated by CHRM1 down-regulation (Figure 3(d)). All the above results uncovered that Germ prevented OA-evoked AD-related pathology by elevating CHRM1 expression.

### Germ leads to the activation of Gq/PLCβ/PKC signaling by enhancing CHRM1 expression in OA-induced PC12 cells

**Figure 4.**
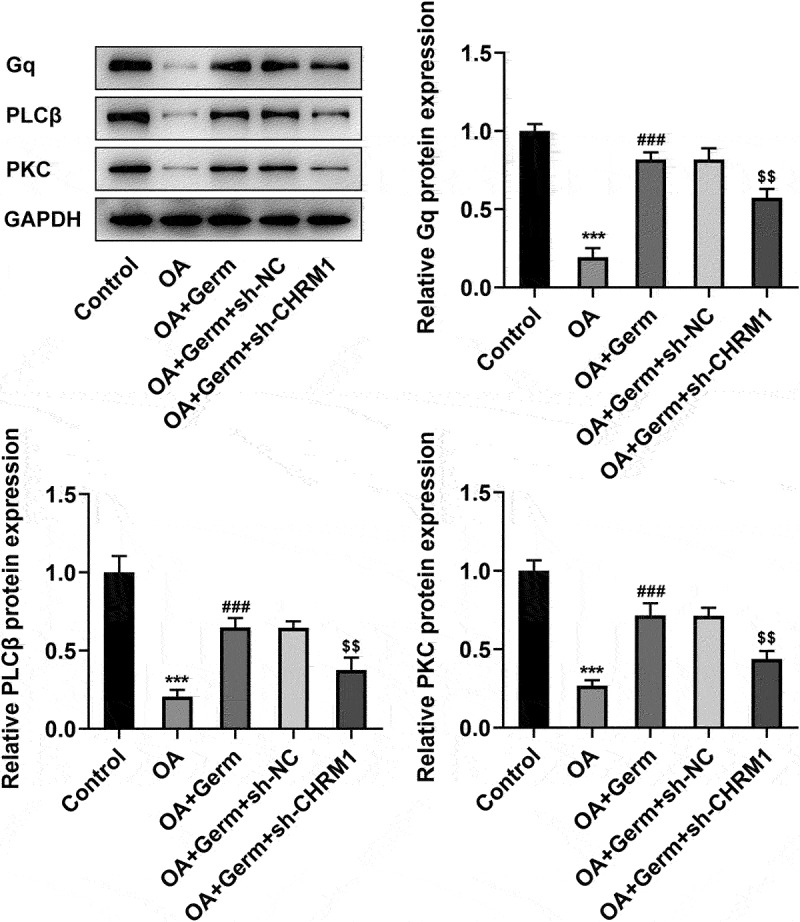
Germ leads to the activation of Gq/PLCβ/PKC signaling by enhancing CHRM1 expression. Western blot was employed to evaluate the protein levels of Gq, PLCβ and PKC. ***P < 0.001 vs. control; ^###^P < 0.001 vs. OA; ^$$^P < 0.01 vs. OA+Germ+sh-NC.

To investigate whether Germ could regulate Gq/PLCβ/PKC signaling by enhancing CHRM1 expression, the expression of proteins in this pathway was determined by Western blot analysis. As shown in Figure 4, the protein levels of Gq, PLCβ and PKC were significantly decreased in OA-treated PC12 cells. After exposed to Germ, Gq, PLCβ and PKC expression was remarkably up-regulated relative to the OA group, while this outcome was partially counteracted by CHRM1 silencing (Figure 4). In conclusion, Germ up-regulates CHRM1 expression to activate the Gq/PLCβ/PKC signaling in OA-induced PC12 cells.

### The inactivation of Gq/PLCβ/PKC signaling attenuates the impacts of Germ on the proliferation and oxidative stress in OA-stimulated PC12 cells

**Figure 5.**
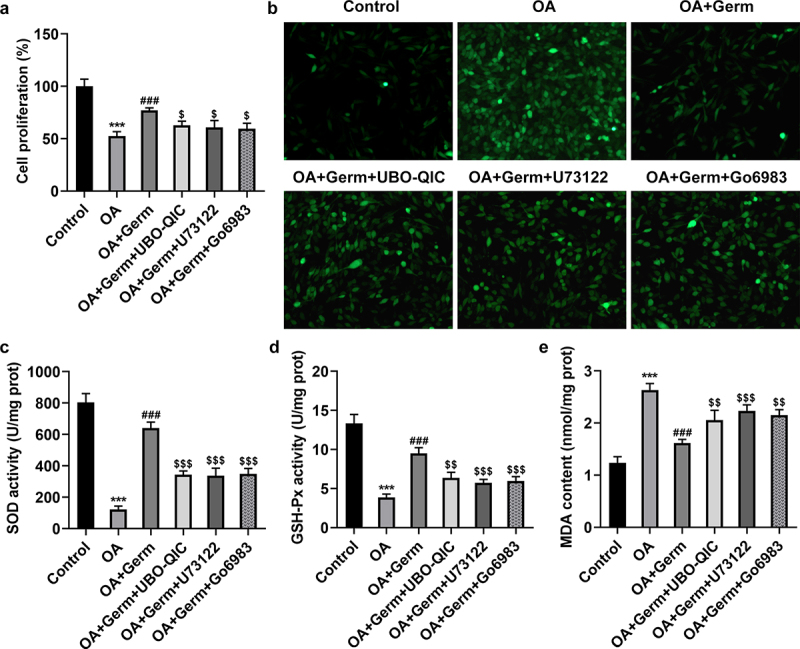
The inactivation of Gq/PLCβ/PKC signaling attenuates the impacts of Germ on the proliferation and oxidative stress in OA-stimulated PC12 cells. (a) CCK-8 assay was used to measure cell proliferation. (b) ROS level was estimated by DCFH-DA assay. (c) SOD, (d) GSH-Px and (e) MDA levels were examined by corresponding kits. ***P < 0.001 vs. control; ^###^P < 0.001 vs. OA; ^$^P < 0.05, ^$$^P < 0.01, ^$$$^P < 0.001 vs. OA+Germ.

To verify the role of Gq/PLCβ/PKC signaling in Germ-mediated neurotoxicity in OA-treated PC12 cells, Gq inhibitor UBO-QIC, PLCβ inhibitor U73122 and PKC inhibitor Go6983 were utilized. CCK-8 assay showed that Germ led to the increase on the proliferation of OA-insulted PC12 cells, while the addition of UBO-QIC, U73122 or Go6983 partially rescued this effect (Figure 5(a)). On the contrary, the suppressive role of Germ in OA-induced ROS levels in PC12 cells was also partially countervailed by UBO-QIC, U73122 or Go6983 (Figure 5(b)). In the same way, UBO-QIC, U73122 or Go6983 partially abrogated the protective role of Germ against OA-mediated oxidative stress in PC12 cells, as evidenced by the up-regulated SOD, GSH-Px and the down-regulated MDA levels in OA-treated PC12 cells caused by Germ (Figure 5(c-e)). All in all, Germ exerts its functions on the proliferation and oxidative stress in OA-stimulated PC12 cells via activating Gq/PLCβ/PKC signaling.

### The inactivation of Gq/PLCβ/PKC signaling weakens the function of Germ on OA-elicited injury of PC12 cells

**Figure 6.**
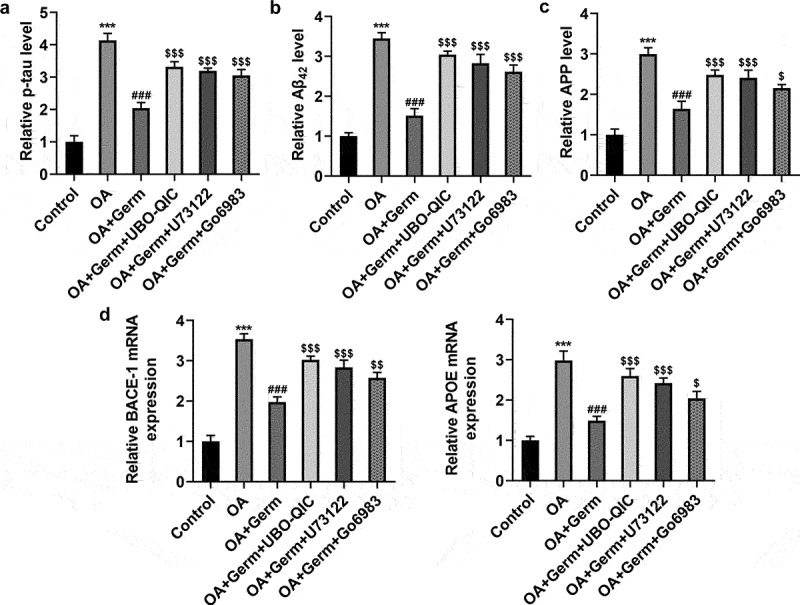
The inactivation of Gq/PLCβ/PKC signaling weakens the function of Germ on OA-elicited injury of PC12 cells. (a) p-tau, (b) Aβ_42_ and (c) APP levels were detected by ELISA assay. (d) BACE-1 and APOE expression was tested by RT-qPCR. ***P < 0.001 vs. control; ^###^P < 0.001 vs. OA; ^$^P < 0.05, ^$$^P < 0.01, ^$$$^P < 0.001 vs. OA+Germ.

The following experiments were used to study whether Germ attenuated the neuron injury in PC12 cells exposed to OA via CHRM1-mediated activation of Gq/PLCβ/PKC signaling. ELISA assay analyzed that Germ exposure notably lessened OA-enhanced p-tau, Aβ_42_ and APP levels in PC12 cells, and this result was partially counteracted by UBO-QIC, U73122 or Go6983 (Figure 6(a-c)). Also, through RT-qPCR analysis, it turned out that OA treatment raised the mRNA levels of BACE-1 and APOE, while UBO-QIC, U73122 or Go6983 partially reversed the declined BACE-1 and APOE mRNA levels in OA-insulted PC12 cells on account of Germ (Figure 6(d)). Taken together, the inactivation of Gq/PLCβ/PKC signaling diminishes the impact of Germ on OA-triggered injury of PC12 cells.

## Discussion

AD represents a complex disorder accompanied by neuron loss, death and damage [4]. OA is a potent polyether marine toxin, which is responsible for diarrhetic shellfish poisoning around the world [33]. A growing body of evidence has supported that OA brings about neurotoxicity in AD, which may provide a novel tool to study AD [34–36]. In this study, OA was applied to establish PC12 cell model of AD and the experimental results revealed that the proliferation of PC12 cells was observably hampered in response to OA.

The excess accumulation of Aβ, which is attributed to the amyloidogenic cleavage of APP, is deemed to display a strong relevance with the pathology of AD, which may further result in neurotoxicity and cognitive impairment [37,38]. Meanwhile, tau phosphorylation is a predominant pathological feature of AD [39–41]. Moreover, tau phosphorylation is triggered under the condition of OA treatment [42]. BACE-1 is a membrane-spanning protein engaged in the hydrolysis of the amyloid precursor protein, thereby being recognized as one of the targets for AD prevention and treatment [43]. Also, APOE remains a key driving factor for AD [44]. Consistent with these findings, it was discovered that the levels of p-tau, Aβ_42_, APP and the mRNA levels of BACE-1 and APOE were all enhanced in PC12 cells after stimulated by OA in this study. Oxidative stress reflects a prooxidant-antioxidant imbalance, as a consequence of which cellular damage is caused [45]. Notably, previous reports have illuminated that oxidative stress participates in the development of AD by promoting Aβ deposition, tau hyperphosphorylation and subsequent neuron loss [46,47]. The generation of excessive ROS is known to be a determinant of oxidative stress [31]. Additionally, SOD and GSH-Px are antioxidant enzymes, while MDA is a marker of oxidative damage [48,49]. Our results also demonstrated that OA treatment down-regulated the levels of SOD and GSH-Px, while up-regulated the level of MDA, which was in agreement with previous findings.

Germ is a well-characterized terpenoid compound extracted from *Curcuma zedoaria* [7]. It is commonly acknowledged as the most effective chemopreventive agents for multiple human diseases. For instance, Wu et al. have elaborated that Germ plays an anti-oxidative role in cerebral ischemia/reperfusion injury [50]. Zhang et al. have validated that Germ impedes the autophagy of PC12 cells to mitigate oxygen–glucose deprivation/reperfusion injury [12]. Nonetheless, the pharmacological effects of Germ against AD are still poorly understood. Through our investigation, single different concentrations of Germ elicited no apparent activities on the proliferation of PC12 cells. However, after exposure to OA, the administration of Germ remarkably enhanced the proliferation of PC12 cells in a concentration-dependent manner. Furthermore, the treatment of Germ (40 μM) ameliorated OA-evoked oxidative stress and injury in PC12 cells, as evidenced by the lessened ROS, MDA, p-tau, Aβ_42_ and APP levels, the reduced BACE-1 and APOE expression and the enhanced SOD and GSH-Px levels in OA-treated PC12 cells caused by Germ treatment.

M1 muscarinic receptor is a G-protein-coupled receptor concerned with multiple pivotal functions of the central and peripheral nervous systems [16,17]. Emerging evidence has testified that M1 mAChR is widely expressed in the central nervous system and activation of M1 muscarinic receptors may influence the occurrence or development of AD via coupling to Gq and activating PLCβ and PKC [20,21]. It is also supposed that dysregulation of cholinergic receptors is ascribed to the amyloid beta pathology [51] and activation of M1 muscarinic receptors also decreases Aβ formation and tau hyperphosphorylation in turn [22,23]. Meanwhile, it is well documented that mAChRs can be activated by turmeric whose genus is the same as *Curcuma zedoaria*. Consistently, CHRM1, Gq, PLCβ and PKC expression was found to be decreased in PC12 cells by OA treatment while enhanced after stimulated by different concentrations of Germ. Functional experiments further manifested that CHRM1 silencing abrogated the promoting role of Germ in the proliferation of OA-induced PC12 cells. In addition, the declined ROS, MDA, p-tau, Aβ42 and APP levels, the decreased BACE-1 and APOE expression and the elevated SOD and GSH-Px levels in OA-treated PC12 cells on account of Germ administration were all reversed by silencing of CHRM1, which suggested that Germ protected against OA-enhanced oxidative stress and neuron injury in PC12 cells through activating CHRM1. What’s more, the up-regulated Gq, PLCβ and PKC protein levels in OA-treated PC12 cells stimulated by OA were all again cut down by deficiency of CHRM1. Besides, Gq inhibitor UBO-QIC, PLCβ inhibitor U73122 and PKC inhibitor Go6983 partially offset the impacts of Germ on the proliferation, oxidative stress and injury in OA-insulted PC12 cells.

## Conclusion

Taken together, Germ alleviated the oxidative stress and neuron injury in OA-induced PC12 cells by up-regulating CHRM1 expression and activating Gq/PLCβ/PKC signaling. To the best of our knowledge, this study is the first to unmask the role of Germ in AD, which may broaden the application area for Germ and offer novel clues for the therapeutic strategy of AD.

## Data Availability

The datasets generated for this study are available on request to the corresponding author.

## Highlights

- Germ elevates CHRM1 and activates Gq/PLCβ/PKC signaling in OA-induced PC12 cells.
- Germ alleviates oxidative stress in OA-induced PC12 cells by activating CHRM1.
- Germ relieves OA-elicited neuron injury of PC12 cells by activating CHRM1.
- Germ attenuates OA-induced PC12 cell damage via CHRM1-mediated Gq/PLCβ/PKC signaling.
